# Analysis of adverse reactions and cost-driving factors during the induction phase of pediatric acute lymphoblastic leukemia

**DOI:** 10.3389/fped.2025.1702696

**Published:** 2025-11-14

**Authors:** Zhonghao Xu, Chenhong Jia, Xiangyu Ding, Xiaohui Chen

**Affiliations:** 1 Department of Personnel and Organization, Hebei Children′s Hospital, Shijiazhuang, Hebei, China; 2 Hebei Provincial Clinical Research Center for Child Health and Disease, Shijiazhuang, Hebei, China; 3 Department of Pharmacy, Hebei Children′s Hospital, Shijiazhuang, Hebei, China

**Keywords:** acute lymphoblastic leukemia, children, induction therapy, adverse reactions, hospitalization costs

## Abstract

**Introduction:**

Acute lymphoblastic leukemia (ALL) is the most common pediatric malignancy and incurs high medical costs due to the complexity and duration of treatment. This study aimed to analyze the incidence of adverse reactions during the induction phase of ALL treatment and identify key factors influencing hospitalization costs.

**Methods:**

A retrospective analysis was conducted on 292 newly diagnosed cases of ALL in children who received induction therapy at Hebei Children's Hospital (2020–2024) under the CCLG-ALL-2018 protocol. Demographic, clinical, laboratory, and hospitalization cost data were collected. Adverse reactions were graded using the Common Terminology Criteria for Adverse Events version 5.0. Hospitalization costs were analyzed using the structural variation method and grey relational analysis. Multivariate linear regression was performed to identify cost-related factors.

**Results:**

The median patient age was 4.9 years (male:female = 6:4). Most cases were B-cell ALL (88.36%), and 69.52% were classified as intermediate risk. Adverse reaction rates were relatively low, including allergic reactions (2.74%), pancreatic toxicity (3.77%), and hepatotoxicity (13.36%). Infections were common, with sepsis occurring in 49.32% and pneumonia in 32.19% of cases. The mean hospitalization costs during the induction phase decreased from 77,690 RMB in 2020 to 56,263 RMB in 2024. Diagnostic fees (37.55%) and medication (30.52%) represented the largest cost components. Hospitalization days, intravenous antifungal medication, tumor lysis syndrome, blood product usage, severe pneumonia infection, surgery performed and discharge yearwere determined to be independent predictors of increased costs.

**Conclusions:**

Adverse reactions and complications markedly burden induction treatment. Optimizing protocols, improving monitoring, and implementing cost-control measures can enhance outcomes and reduce costs. Larger sample sizes and socioeconomic variables should be included in future research.

## 1 Introduction

Leukemia is one of the most common malignancies in children, with acute lymphoblastic leukemia (ALL) being the most prevalent subtype. In China, between 2018 and 2020, the incidence rate of ALL in children and adolescents was 21.96 per million, accounting for 55.82% of all leukemia cases (total leukemia incidence: 39.34 per million) (1). In 2021, although China's age-standardized incidence rate and age-standardized death rate for leukemia were lower than those in the United States, the incidence and mortality rates of ALL in China were relatively higher (2).

In recent years, continuous optimization of chemotherapy regimens and the widespread application of comprehensive treatment methods have significantly improved the long-term survival rate of children with ALL (3). Induction remission chemotherapy, the primary treatment modality for children with ALL, achieves complete remission in over 95% of patients through combination therapy with vincristine, corticosteroids, asparaginase, and anthracyclines, demonstrating remarkable efficacy (4). However, the complexity and prolonged duration of treatment contribute to persistently high overall treatment costs. This issue is reflected in studies from various regions in China. For example, a study from the Children's Hospital affiliated with Chongqing Medical University showed that the average total medical cost for the induction remission phase of newly diagnosed children was approximately 60,000 RMB (5). If more subsequent treatment phases are included, the cost exceeds 200,000 RMB (6, 7). Internationally, treatment costs for children with ALL remain high compared with those in China. One study reported varying costs depending on the treatment regimen and patient characteristics, but overall costs were substantial, with the highest expenses typically incurred during the first year and at the end of life (8). The use of CAR-T cell immunotherapy for the treatment of relapsed or refractory ALL can cost several hundred thousand U.S. dollars (9).

During the induction remission phase, patients often experience treatment-related adverse reactions, which adversely affect treatment outcomes and prognosis (3, 10). Severe adverse events (SAEs) further increase the risk of treatment-related mortality (TRM), with reported incidence rates of 7.6% for SAEs and 3.4% for TRM in pediatric ALL chemotherapy (10). The rapid reduction of leukemia cells caused by induction remission chemotherapy may also induce severe complications such as tumor lysis syndrome (TLS), myelosuppression, and systemic infection, which are closely associated with treatment failure and patient death (11). In addition, the clinical impact of multiple drug-related specific toxicities is particularly prominent: As a key drug in the induction therapy of ALL, PEG-asparaginase can cause adverse reactions (e.g., severe pancreatitis, coagulation dysfunction) that may directly interrupt the treatment process and require intensive intervention measures; Although anthracyclines have definite efficacy, childhood cancer survivors who receive this class of drugs face a significantly increased risk of cardiovascular diseases in adulthood, especially cardiomyopathy and coronary artery disease identified in long-term follow-up (12). The health burden caused by these diseases far exceeds the cost of acute-phase treatment. Moreover, neurotoxicity induced by vincristine can directly impair patients’ quality of life, and rehabilitation therapy is often required to relieve symptoms. A systematic review on the cost-effectiveness analysis of anticancer drugs shows that underestimating AE-related costs leads to deviations in cost-effectiveness conclusions in nearly half of the studies (13), highlighting the necessity of incorporating the characteristics of adverse events (AEs) into economic evaluations.

Moreover, year-to-year variations in these costs may arise from factors such as advancements in medical technology, changes in drug prices, and adjustments to medical insurance policies (14). Against this background, the present study aims to provide references for clinical practice by analyzing the incidence of key AEs during the induction phase of childhood ALL and their impact on hospitalization costs.

## 2 Methods

### 2.1 Ethics approval and consent to participate

The Ethics Committee of the Hebei Children's Hospital (Ethical Review Number: 202407–63) approved the study protocol. The requirement for informed consent was waived due to the retrospective and anonymized nature of the data.

### 2.2 Study population

This retrospective study included patients diagnosed with ALL or lymphoblastic lymphoma between January 2020 and December 2024, identified through the Medical Record Management System of Hebei Children's Hospital.

Inclusion criteria were as follows:

1. Age 1–18 years and treated according to the CCLG-ALL-2018 protocol (15)
2. Definitive diagnosis confirmed *via* Morphology–Immunophenotype–Cytogenetics–Molecular biology (MICM) analysis
3. Receipt of induction therapy with VDLP/VDLD regimens comprising prednisone/dexamethasone, vincristine, daunorubicin, and PEG-asparaginase. For patients with lymphoblastic lymphoma, only those who received the aforementioned VDLP/VDLD induction regimens were included.

### 2.3 Data collection

Clinical data collected included:

  Demographics: age, sex, weight, height, and body surface area.

  Disease characteristics: immunophenotype, risk stratification, and central nervous system (CNS) status.

  Laboratory results: baseline and peak/trough values during induction.

  Adverse reactions recorded during induction.

Total hospitalization costs (adjusted to 2020 constant RMB), including comprehensive medical services (general services, treatment, and nursing); diagnostics (pathology, laboratory, and imaging); therapeutics (non-surgical and surgical interventions); and pharmaceuticals, blood products, and medical consumables.

### 2.4 Assessment of adverse reactions

Adverse events were graded using the National Cancer Institute's Common Terminology Criteria for Adverse Events (CTCAE; version 5.0) (16). Key adverse events monitored included allergic reactions, pancreatitis, hepatotoxicity, coagulation disorders, myelosuppression, infections, and organ-specific toxicities.

### 2.5 Statistical analysis

Normally distributed data are expressed as mean ± SD, while non-normally distributed data are expressed as median (P25, P75). Cost-related factors were examined using Mann–Whitney U or Kruskal–Wallis tests, and significant variables were entered into multivariate analysis with total costs as the dependent variable. Cost structure was assessed using the structural variation value (VSV), degree of structural variation (DSV), and contribution rate (17). Grey correlation analysis was applied to assess associations between cost components and total expenses (17). Statistical significance was set at *P* < 0.05. Data were analyzed using Excel 2019, SPSS 26.0, and GraphPad Prism 10.1.2. Excel 2019 was used to collate baseline patient data, calculate the incidence of adverse reactions, compute structural variation indicators of hospitalization costs, and perform grey relational analysis to identify cost-related associations. SPSS 26.0 was used to determine the distribution of continuous variables, conduct univariate analysis of cost-influencing factors, and construct a multivariate linear regression model to identify independent predictors of hospitalization costs. GraphPad Prism 10.1.2 was used to create charts to display the trends and compositions of annual average hospitalization costs and to visualize the key factors driving higher costs identified by regression analysis.

## 3 Results

### 3.1 Patient characteristics

A total of 292 children with newly diagnosed ALL were included in this study. The median age was 4.9 years (range: 0.7–18.6 years), with a male-to-female ratio of approximately 6:4. Most patients had B-cell ALL (88.36%, *n* = 258), and 69.52% (*n* = 203) were classified as intermediate-risk. The most common symptoms at admission were fever (56.51%, *n* = 165) and abnormal blood counts (23.63%, *n* = 69). CNS1 was observed in 82.53% (*n* = 241) of cases. By day 33 of induction therapy, 85.27% (*n* = 249) of patients had minimal residual disease <0.01%, and 99.32% (*n* = 290) achieved good bone marrow remission (Table 1).

**Table 1:** Baseline data and characteristics.

Number of patients	*n* = 292
Age (years), median (range), *n* (%)	4.9 (0.7–18.6)
<1	1 (0.34)
≥1, <10	239 (81.85)
≥10	52 (17.81)
Body weight (kg), median (range)	18 (8.0–87.5)
Body height (cm), median (range)	107 (65–179)
Body surface area (m^2^), median (range)	0.73 (0.38–1.83)
Sex, *n* (%)	
Male	167 (57.19)
Female	125 (42.81)
Length of hospital stay (days), median (range)	32 (15–93)
Clinical Characteristics, *n* (%)	
Mediastinal mass	5 (1.71)
Testicular infiltration	3 (1.03)
Bone and joint infiltration	1 (0.34)
Central nervous system status, *n* (%)	
CNS1	241 (82.53)
CNS2	10 (3.42)
CNS3	41 (14.04)
Initial Symptoms, *n* (%)	
Fever	165 (56.51)
Abnormal blood count	69 (23.63)
Pale or anemic	67 (22.95)
Joint or bone pain	57 (19.52)
Bruises or bleeding	30 (10.27)
Respiratory symptoms	29 (9.93)
Fatigue	28 (9.60)
Abdominal discomfort	22 (7.53)
Mass	18 (6.16)
Lymphadenopathy	9 (3.08)
Immunophenotyping, *n* (%)	
B-ALL	258 (88.36)
T-ALL	24 (8.22)
T-ALL/LBL	7 (2.40)
B-ALL/LBL	3 (1.03)
Risk group, *n* (%)	
Low risk (LR)	38 (13.01)
Intermediate risk (IR)	203 (69.52)
High risk (HR)	51 (17.47)
Induction therapy response, *n* (%)	
MRD (%) at D15	
<0.01	99 (33.90)
≥0.01, <0.1	19 (6.51)
≥0.1, <1	75 (25.68)
≥1, <10	63 (21.58)
≥10	36 (12.33)
Blasts and immature cells (%) at D15	
<5	262 (89.73)
≥5, <20	13 (4.45)
≥20	17 (5.82)
MRD (%) at D33	
<0.01	249 (85.27)
≥0.01, <0.1	19 (6.51)
≥0.1, <1	14 (4.79)
≥1, <10	6 (2.05)
≥10	4 (1.37)
Blasts and immature cells (%) at D33	
<5	290 (99.32)
≥5, <20	1 (0.34)
≥20	1 (0.34)

B-ALL, B-cell acute lymphoblastic leukemia; T-ALL, T-cell acute lymphoblastic leukemia; T-ALL/LBL, T-lymphoblastic lymphoma/leukemia; B-ALL/LBL, B-lymphoblastic lymphoma/leukemia; MRD, minimal residual disease.

### 3.2 Adverse reactions

Allergic events were uncommon, occurring in 2.74% (*n* = 8) of cases. Among these, two cases involved allergic shock, one attributed to PEG-asparaginase and the other to plasma, while the remaining cases presented as skin rashes caused by other drugs. The incidence of pancreatitis was 3.77% (*n* = 11), primarily manifesting as acute pancreatitis. Hepatic dysfunction was more frequent, with 13.36% (*n* = 39) of patients experiencing grade 2 or higher increases in transaminases, 5.14% (*n* = 15) reaching grades 3–4, and 27.05% (*n* = 79) developing hyperbilirubinemia. Coagulation disorders were prevalent, with 91.78% (*n* = 268) experiencing grade 3 or higher reductions in fibrinogen and two cases of thrombotic events. Bone marrow suppression was observed, with grade 3–4 neutropenia in 56.51% (*n* = 165), grade 3–4 anemia in 26.37% (*n* = 77), and grade 3–4 thrombocytopenia in 8.90% (*n* = 26) of patients. Infection complications included sepsis (49.32%, *n* = 144) and pneumonia (32.19%, *n* = 94). Other adverse reactions included myocardial injury (8.22%, *n* = 24), vincristine-related neurotoxicity (7.88%, *n* = 23), and tumor lysis syndrome (2.05%, *n* = 6) (Table 2).

**Table 2:** Incidence of adverse reactions.

Adverse reaction	*N* (%)
Allergic reactions	
Allergic skin rash	6 (2.05)
Anaphylactic shock	2 (0.68)
Pancreatic toxicity	
Acute pancreatitis	11 (3.77)
Hypertriglyceridemia	1 (0.34)
Hyperglycemia	13 (4.45)
Hypoglycemia	6 (2.05)
Hepatotoxicity	
ALT/AST increased (Grade ≥2)	39 (13.36)
Grade 3–4	15 (5.14)
TBIL/DBIL increased (Grade ≥2)	79 (27.05)
Grade 3–4	7 (2.40)
Hypoalbuminemia (Grade ≥2)	45 (15.41)
Coagulation disorders	
Prolonged APTT (Grade ≥2)	37 (12.67)
Grade 3	9 (3.08)
Fibrinogen decreased (Grade ≥2)	275 (94.18)
Grade 3–4	268 (91.78)
Thrombosis	2 (0.68)
Peripheral blood decrease	
NE decreased (Grade ≥2)	167 (57.19)
Grade 3–4	165 (56.51)
HGB decreased (Grade ≥2)	121 (41.44)
Grade 3–4	77 (26.37)
PLT decreased (Grade ≥2)	47 (16.10)
Grade 3–4	26 (8.90)
Infection	
Pneumonia	94 (32.19)
Sepsis	144 (49.32)
Oral mucositis	63 (21.58)
Other infections	12 (4.11)
Gastrointestinal discomfort	
Diarrhea	33 (11.30)
Nausea and vomiting	13 (4.45)
Others	
Myocardial injury	24 (8.22)
Neurotoxicity	23 (7.88)
Hypertension	2 (0.68)
Tumor Lysis Syndrome	6 (2.05)
Electrolyte disturbances	37(12.67)

Patients with abnormal baseline values were excluded. The total number of patients was 292. All aforementioned adverse reactions occurred following the administration of the first dose of PEG-asparaginase. Hyperglycemia was defined as fasting blood glucose >7.0 mmol/L or random blood glucose >11.1 mmol/L, while hypoglycemia was defined as fasting blood glucose <2.8 mmol/L (34).

NE, neutrophil; HBG, hemoglobin; PLT, platelet; ALT, alanine aminotransferase; AST, aspartate aminotransferase; TBIL, total bilirubin; DBIL, direct bilirubin; APTT, activated partial thromboplastin time.

### 3.3 Hospitalization costs

**Figure 1:**
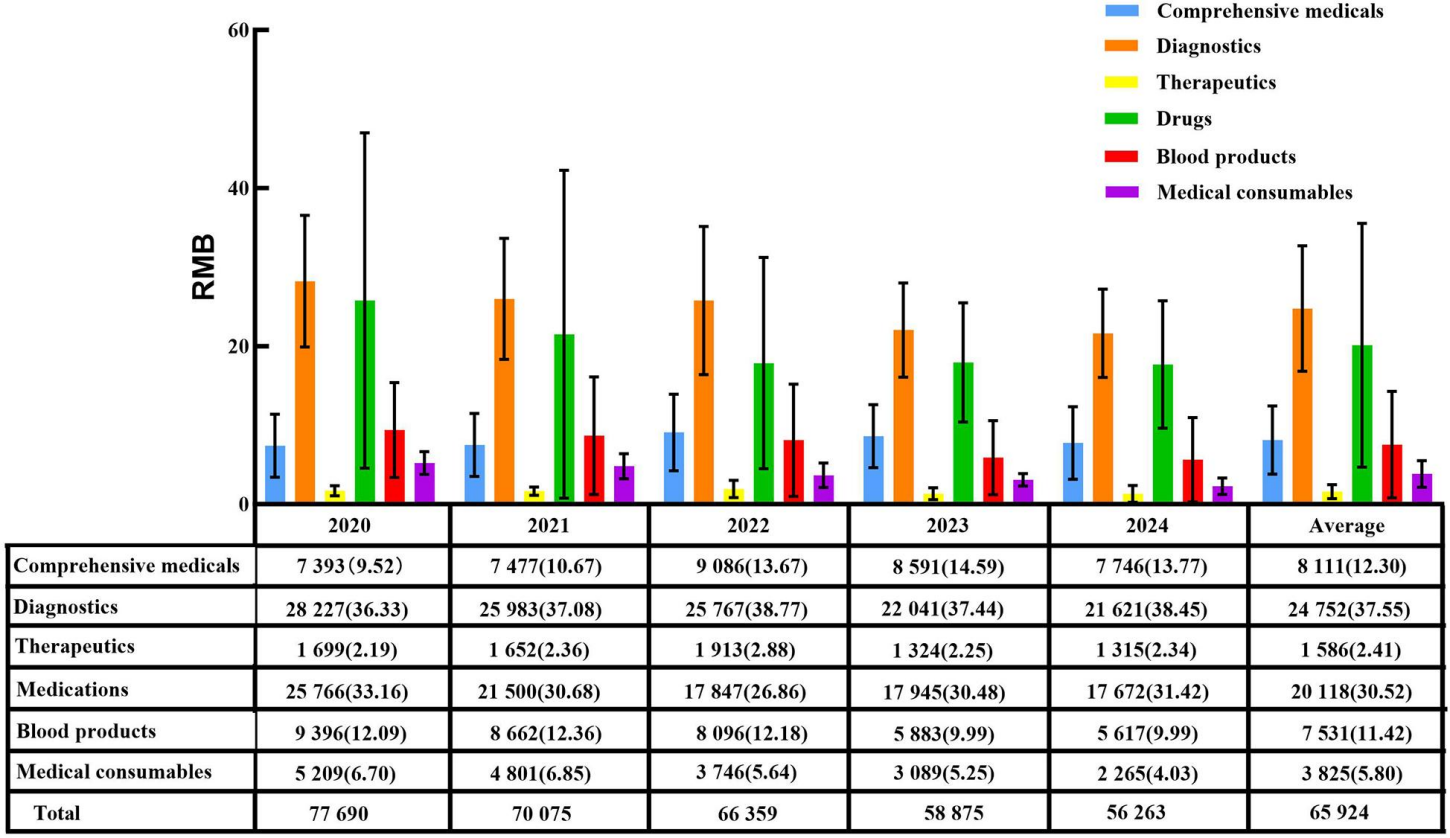
Average hospitalization costs and their proportions during the induction phase in patients with ALL from 2020 to 2024 [RMB(%)].

From 2020–2024, per capita hospitalization costs for patients with ALL during the induction phase continuously decreased from 77,690 RMB to 56,263 RMB, with an overall mean of 65,924 RMB. Diagnostic fees (37.55%) and medication (30.52%) constituted the largest proportions of total costs (Figure 1). The main contributors to structural change were, in descending order, comprehensive medical services (32.64%), consumables (20.52%), diagnostic fees (16.20%), and blood products (16.14%) (Table 3). Grey relational analysis showed that diagnostic fees had the highest correlation with total costs (0.88), followed by drugs (0.79) (Table 4).

**Table 3:** Distribution of contribution rates from structural variations (2020–2024, %).

Cost category	2020–2021		2021–2022		2022–2023		2023–2024		2020–2024	
	VSV	Rates	VSV	Rates	VSV	Rates	VSV	Rates	VSV	Rates
Comprehensive medicals	1.15	23.23	3.00	28.77	0.92	10.12	−0.82	20.04	4.26	32.64
Diagnostics	0.75	15.02	1.69	16.24	−1.33	14.68	1.01	24.70	2.11	16.20
Therapeutics	0.17	3.42	0.52	5.00	−0.63	6.93	0.09	2.18	0.15	1.16
Medication	−2.48	50.00	−3.83	36.66	3.63	39.88	0.94	23.12	−1.76	13.34
Blood products	0.27	5.37	−0.18	1.70	−2.19	24.10	0.00	0.09	−2.11	16.14
Medical consumables	0.15	2.96	−1.21	11.64	−0.39	4.30	−1.22	29.87	−2.68	20.52
DSV	4.97	—	10.44	—	9.09	—	4.08	—	13.05	—

VSV, structural variation value; DSV, degree of structural variation.

**Table 4:** New grey relational analysis of hospitalization costs (2020–2024).

Cost category	Correlation coefficient					Correlation degree	Correlation ranking
	2020	2021	2022	2023	2024		
Comprehensive medicals	0.34	0.50	0.53	0.44	0.56	0.47	5
Diagnostics	0.79	0.93	0.81	1.00	0.88	0.88	1
Therapeutics	0.57	0.89	0.40	0.71	0.87	0.69	3
Medication	0.58	0.99	0.53	1.00	0.86	0.79	2
Blood products	0.67	0.61	0.68	0.55	0.56	0.61	4
Medical consumables	0.42	0.41	0.84	0.62	0.34	0.53	6

### 3.4 Factors influencing costs

**Figure 2:**
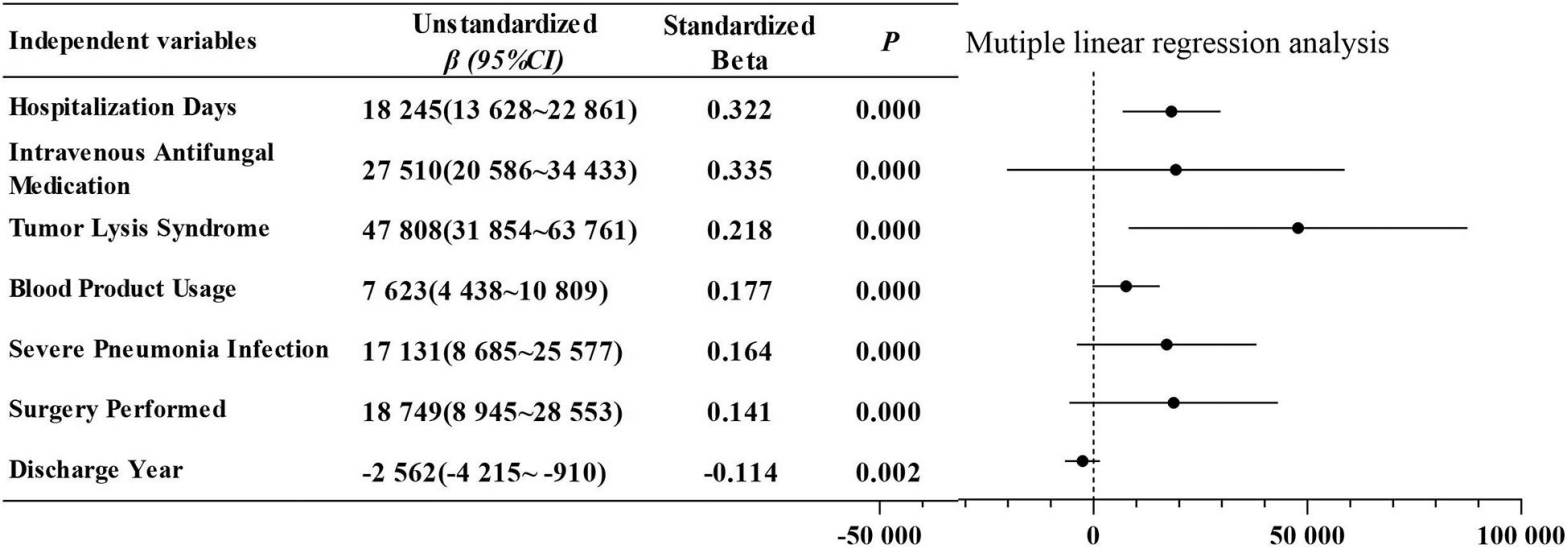
Key factors driving higher hospitalization costs in pediatric ALL induction phase.

Univariate analysis indicated that hospitalization costs during the induction phase were influenced by several factors, including hospitalization days, discharge year, payment method, surgery performed, blood product usage intravenous antifungal medication, special-grade antibioticsusage, severe pneumonia infection, admission to the intensive care unit, tumor lysis syndrome, pancreatitis, and hepatic dysfunction (Table 5). In the subsequent multivariate analysis, which included independent variables identified as significantly different from the univariate analysis, hospitalization days, intravenous antifungal medication, tumor lysis syndrome, blood product usage, severe pneumonia infection, surgery performed and discharge yearwere determined to be independent predictors of increased costs (Figure 2). This model yielded an adjusted *r*^2^ of 0.619 (F = 68.5, *P* < 0.001). The Durbin–Watson statistic was 2.251, indicating independence of residuals, and the variance inflation factors ranged from 1.040–1.403, suggesting no multicollinearity among variables.

**Table 5:** Univariate analysis of total hospitalization costs and variable assignments during the induction phase in patients with ALL.

Code	Variable and assignments	*N* (%)	Total cost median (RMB)	U/H	*P*-Value
X_1_	Sex			9,850.000	0.411
	“Male” = 1	167 (57.19)	55,412 (47,206, 73,715)		
	“Female” = 2	125 (42.81)	58,393 (47,849, 72,335)		
X_2_	Age (years)			6,212.000	0.960
	“<10” = 1	240 (82.19)	56,397 (47,467, 74,096)		
	“≥10” = 2	52 (17.81)	57,020 (47,495, 70,190)		
X_3_	Body surface area (m^2^)			0.145	0.930
	“<0.8” = 1	165 (56.51)	55,555 (48,065, 72,835)		
	“≥0.8, <1.2” = 2	82 (28.08)	56,844 (47,444, 79,042)		
	“≥1.2” = 3	45 (15.41)	57,568 (45,589, 71,538)		
X_4_	Hospitalization days (days)			122.442	0.000***
	“≤28” = 1	112 (38.36)	47,470 (40,784, 53,454)		
	“>28, ≥56” = 2	169 (57.88)	65,733 (54,270, 84,555)		
	“>56” = 3	11 (3.77)	147,159 (93,875, 183,860)		
X_5_	Discharge year (y)			19.491	0.001**
	“2020” = 1	61 (20.89)	66,619 (50,325, 93,879)		
	“2021” = 2	50 (17.12)	58,542 (47,203, 82,788)		
	“2022” = 3	64 (21.92)	55,027 (48,275, 73,550)		
	“2023” = 4	66 (22.60)	55,185 (46,754, 66,464)		
	“2024” = 5	51 (17.47)	51,509 (45,691, 57,850)		
X_6_	Payment Method			3,726.000	0.039*
	“Self-paying” = 1	37 (12.67)	65,640 (48,108, 81,324)		
	“Medical insurance” = 2	255 (87.33)	55,412 (47,365, 70,930)		
X_7_	Surgery performed			1,120.000	0.000***
	“No” = 0	275 (94.18)	55,412 (47,365, 70,930)		
	“Yes” = 1	17 (5.82)	74,148 (63,730, 113,337)		
X_8_	Blood product usage			37.043	0.000***
	“None or single type” = 1	57 (19.52)	47,488 (40,673, 54,743)		
	“Two types” = 2	133 (45.55)	56,668 (47,441, 72,392)		
	“More than two types” = 3	102 (34.93)	61,551 (52,551, 87,995)		
X_9_	Intravenous antifungal medication			1,235.000	0.000***
	“No” = 0	241 (82.53)	52,916 (46,380, 63,134)		
	“Yes” = 1	51 (17.47)	96,028 (70,930, 138,997)		
X_10_	Special-grade antibiotic usage			72.843	0.000***
	“None or single type” = 1	198 (67.81)	51,472 (44,946, 61,965)		
	“Two types” = 2	60 (20.55)	65,936 (53,324, 82,056)		
	“More than two types” = 3	34 (11.64)	96,202 (71,542, 123,493)		
X_11_	Severe pneumonia infection			896.000	0.000***
	“No” = 0	263 (90.07)	54,509 (47,129, 67,432)		
	“Yes” = 1	29 (9.93)	103,130 (79,417, 134,620)		
X_12_	ICU			335.000	0.003**
	“No” = 0	285 (97.60)	55,556 (47,425, 71,819)		
	“Yes” = 1	7 (2.40)	106,647 (70,930, 146,339)		
X_13_	Tumor lysis syndrome			135.000	0.000***
	“No” = 0	286 (97.95)	55,550 (47,444, 71,324)		
	“Yes” = 1	6 (2.05)	125,650 (89,830, 157,310)		
X_14_	Pancreatitis			358.000	0.000***
	“No” = 0	281 (96.23)	55,464 (47,376, 70,950)		
	“Yes” = 1	11 (3.77)	101,040 (70,930, 138,997)		
X_15_	Liver function impairment			2,818.000	0.039*
	“No” = 0	264 (90.41)	55,460 (47,318, 71,120)		
	“Yes” = 1	28(9.59)	68,120 (49,677, 90,952)		
Y	Total cost (RMB)				

Blood products include frozen plasma, suspended red blood cells, cryoprecipitate, platelets, and others. Special-grade antibiotics refer to those with a high risk of severe adverse reactions, are prone to bacterial resistance, are expensive, or have limited clinical data. Their use requires consultation with experts and strict adherence to a tiered management system. Examples include meropenem, imipenem, vancomycin, teicoplanin, linezolid, tigecycline, polymyxin, caspofungin, posaconazole, and amphotericin B. All quantitative data are represented by P50 (P25, P75).

* *P* < 0.05.

** *P* < 0.01.

*** *P* < 0.001.

## 4 Discussion

Adverse reactions and complications during pediatric ALL treatment affect patient compliance and quality of life, and increase medical resource utilization and economic burden. This study retrospectively analyzed induction-phase treatment data of 292 children with ALL at Hebei Children's Hospital between 2020 and 2024. Adverse reactions were graded according to CTCAE 5.0, and abnormalities related to chemotherapeutic agents were systematically quantified. Multiple linear regression was used to clarify the association between chemotherapy-related adverse reactions and complications and hospitalization costs, providing a basis for cost control and treatment optimization.

This study found that the incidence and severity of adverse reactions associated with PEG-asparaginase should be closely monitored during the induction phase of treatment. Coagulation disorders were particularly prominent, with 91.78% of patients experiencing grade 3 or higher fibrinogen reduction. This finding is consistent with PEG-asparaginase inhibiting hepatic protein synthesis, leading to reduced coagulation factor production (18). Although only two cases of thrombosis occurred in this study, the findings highlight a dual risk of “bleeding-thrombosis” during ALL treatment, necessitating dynamic monitoring of coagulation indicators. Additionally, the incidence of acute pancreatitis was 3.77%. Early clinical manifestations included gastrointestinal symptoms such as nausea, vomiting, and abdominal pain, accompanied by elevated serum amylase. In this study, only one case of anaphylactic shock was associated with PEG-asparaginase, which improved after symptomatic treatment. This may be attributed to the low immunogenicity of pegaspargase, resulting in a low incidence of allergic reactions (19). Abnormal blood glucose levels were observed in 19 patients, which were strongly associated with the use of glucocorticoids in the chemotherapy regimen, although the effects of PEG-asparaginase on insulin secretion and carbohydrate metabolism could not be ruled out (20). These patients generally did not require hypoglycemic therapy, as their blood glucose levels gradually normalized after chemotherapy.

Adverse reactions to other chemotherapeutic drugs should not be overlooked. The incidence of vincristine-related neurotoxicity was 7.88% and may result from the disruption of normal microtubule dynamics, endothelial damage in nerve cells, and mitochondrial injury (21). Clinical manifestations include sensory abnormalities, muscle weakness, and intestinal obstruction, all of which were observed in this study, and they affect patients’ quality of life. The incidence of daunorubicin-induced myocardial injury was 8.22%, associated with the cardiotoxicity of anthracycline drugs. Severe cases can present with heart failure, highlighting the need for regular monitoring of cardiac function indicators. Neurotoxicity and cardiotoxicity may increase subsequent treatment costs for patients, but this could not be demonstrated in this study.

Infectious complications pose the most substantial safety concern during the induction phase. In this study, the incidence of sepsis reached 49.32%, while pneumonia occurred in 32.19% of cases, both closely linked to immune dysfunction resulting from myelosuppression. Grade 3–4 neutropenia was observed in 56.51% of patients. Since neutrophils are the primary effectors of innate immunity, their deficiency (less than 0.5 × 10^9^ /L) substantially increases the risk of infection (22). Moreover, the spectrum of pathogens was diverse, including bacteria (e.g., *Staphylococcus epidermidis*, *Klebsiella pneumoniae*), fungi (e.g., *Aspergillus fumigatus*, *Candida albicans*), and viruses (e.g., EB virus, cytomegalovirus). Although identifying pathogens is crucial for guiding subsequent treatment, successful isolation is not always achieved, posing a challenge to clinical treatment (23, 24).

While the clinical impact of adverse reactions is well recognized, the economic burden and cost structure of pediatric ALL treatment during the induction phase also warrant detailed analysis. Diagnostic (37.55%) and drug (30.52%) costs account for over 60% of total expenses, making them the primary cost drivers. The high proportion of diagnostic costs reflects the complexity of ALL diagnosis, which requires a comprehensive MICM system and multiple bone marrow aspirations, flow cytometry, and imaging examinations to clarify disease subtype and assess treatment response. The accumulation of these examinations directly increases diagnostic costs. Grey relational analysis further demonstrated that diagnostic costs had the highest correlation with total costs (0.88), confirming their critical impact on overall expenses.

In recent years, reforms in medical insurance payment methods, mainly the diagnosis-related groups (DRG) payment and disease-based payment (DIP) models (25), have shown outstanding performance in optimizing cost structure and improving the efficiency of medical resource utilization. From 2020–2024, the total hospitalization costs during the induction phase showed a continuous decline, aligning with the trend of decreasing average hospitalization costs in China (14, 17, 26). This finding suggests that cost-control measures may have positively contributed to reducing the treatment burden for patients with ALL. The structural variation analysis of cost composition showed that comprehensive medical service costs had the highest contribution rate (32.64%) and continued to grow, reflecting rational adjustments in labor costs for medical services. In contrast, the contribution rates of drugs (13.34%) and consumables (20.52%) declined, largely owing to national policies, such as the Zero Markups Policy for Drugs, Centralized Volume-based Procurement for Drugs, and the informatized supervision of consumables (27–30). Blood costs accounted for 16.14% of total expenses, with a correlation coefficient of 0.61, reflecting the high demand for blood products in ALL treatment. This highlights the need for rational utilization and cost control of blood products.

Compared with other diseases, the high proportion of diagnostic and drug costs identified in this study is similar to that of oral cancer. However, it differs significantly from gynecological tumors, where surgical and laboratory fees dominate expenses, and from cerebral infarction, where drug and rehabilitation costs are the primary contributors (17, 31, 32). This variation underscores the strong influence of disease-specific diagnostic and treatment characteristics on cost structure.

Further analysis showed that adverse reactions and complications not only present clinical challenges but also serve as key drivers of hospitalization costs through both direct and indirect mechanisms.

Direct effects are mainly reflected in additional medical interventions caused by adverse reactions. Previous studies have confirmed that the total cost for pediatric patients with adverse events during ALL induction therapy is over three times higher than those without (33), a pattern further validated in this study. For example, infectious complications lead to increased use of special-grade antibiotics and intravenous antifungal drugs. In this study, patients receiving intravenous antifungal drugs had hospitalization costs of 96,028 RMB, which was 1.81 times higher than those not receiving such therapy (52,916 RMB). Coagulation abnormalities were also common, with 91.78% of patients experiencing grade 3 or higher fibrinogen reduction, and anemia or thrombocytopenia caused by myelosuppression required additional blood product transfusions. The median cost for patients using more than two types of blood products (61,551 RMB) was significantly higher than that for those not using or using only one type (47,488 RMB). Similarly, tumor lysis syndrome (incidence, 2.05%) was associated with increased costs, with a median cost of 125,650 RMB, as it often accompanies electrolyte disturbances and acute renal failure, requiring intensified symptomatic treatment and monitoring.

Indirect effects are primarily manifested in prolonged hospital stays due to adverse reactions, which increase comprehensive medical service fees and diagnostic costs. In this study, the median hospital stay was 32 days. Patients with hospital stays exceeding 56 days incurred costs of 147,159 RMB, which was 3.1 times higher than those with hospital stays of less than 28 days (47,470 RMB). Reasons for extended hospital stays include SAEs (e.g., severe pneumonia and pancreatitis) requiring long-term symptomatic treatment, poor infection control necessitating extended antimicrobial treatment cycles, and adverse reactions causing interruptions or delays in chemotherapy.

Univariate analysis in this study revealed that coagulation abnormalities, pancreatitis, and liver dysfunction significantly impacted medical costs. Among these factors, coagulation abnormalities were particularly prominent due to the need for repeated transfusions of plasma and blood products. Treatment measures for pancreatitis, including fasting, gastrointestinal decompression, and nutritional support, not only increased medical costs but also prolonged hospital stays and elevated the infection rate. Liver dysfunction also contributed to extended hospital stays. Since patients often experience multiple adverse reactions or complications simultaneously due to their underlying disease and chemotherapy, these factors may not appear as independent risk factors in multivariate analysis. Therefore, although chemotherapy-related adverse reactions are not independent risk factors, they still contribute to the increase in medical costs. Additionally, they may raise TRM and lead to chemotherapy interruption. In conclusion, optimizing chemotherapy regimens and early identification of adverse risks to effectively reduce additional medical costs hold significant clinical and economic importance.

This study has some limitations. First, data were obtained from a single center, which may have introduced regional bias. Second, socio-economic factors such as patient family income and medical insurance reimbursement ratios were not included, potentially limiting the assessment of actual out-of-pocket expenses. Lastly, this study focused solely on the induction remission phase and did not examine long-term treatment cost trends. Future research should expand the sample size, incorporate multi-center data with patient socio-economic characteristics, and further refine the cost impact factor model to provide more accurate references for optimizing medical resource allocation for patients with ALL.

## 5 Conclusions

Adverse reactions and treatment-related complications contribute to both clinical risks and economic burdens in pediatric ALL induction therapy. Implementing targeted strategies to reduce severe infections, optimize supportive care, and streamline diagnostics could improve outcomes while controlling costs. Additionally, targeted prevention and early management of adverse events, along with ongoing health-policy reforms such as DRG/DIP and centralized procurement, are urgently needed to mitigate both clinical toxicity and economic burdens in pediatric ALL induction therapy.

## Data Availability

The original contributions presented in the study are included in the article/Supplementary Material, further inquiries can be directed to the corresponding author/s.
